# Effect of l-arginine on energy metabolism, skeletal muscle and brown adipose tissue in South Asian and Europid prediabetic men: a randomised double-blinded crossover study

**DOI:** 10.1007/s00125-018-4752-6

**Published:** 2018-10-30

**Authors:** Mariëtte R. Boon, Mark J. W. Hanssen, Boudewijn Brans, Cindy J. M. Hülsman, Joris Hoeks, Kimberly J. Nahon, Charlotte Bakker, Jan B. van Klinken, Bas Havekes, Gert Schaart, Ingrid M. Jazet, Patrick C. N. Rensen, Wouter D. van Marken Lichtenbelt

**Affiliations:** 1 Dept of Human Biology & Human Movement Sciences, NUTRIM School for Nutrition and Translational Research in Metabolism, Maastricht University Medical Center, Maastricht, the Netherlands; 2 Dept of Medicine, Division of Endocrinology, post zone C7Q, Leiden University Medical Center, P. O. Box 9600, 2300 RC Leiden, the Netherlands; 3 Einthoven Laboratory for Experimental Vascular Medicine, Leiden University Medical Center, Leiden, the Netherlands; 4 Dept of Nuclear Medicine, Maastricht University Medical Center, Maastricht, the Netherlands; 5 Dept of Human Genetics, Leiden University Medical Center, Leiden, the Netherlands; 6 Dept of Internal Medicine, Division of Endocrinology, Maastricht University Medical Center, Maastricht, the Netherlands

**Keywords:** Brown adipose tissue, l-Arginine, Nitric oxide, Skeletal muscle, South Asian

## Abstract

**Aims/hypothesis:**

Individuals of South Asian origin are at increased risk of developing type 2 diabetes mellitus and associated comorbidities compared with Europids. Disturbances in energy metabolism may contribute to this increased risk. Skeletal muscle and possibly also brown adipose tissue (BAT) are involved in human energy metabolism and nitric oxide (NO) is suggested to play a pivotal role in regulating mitochondrial biogenesis in both tissues. We aimed to investigate the effects of 6 weeks of supplementation with l-arginine, a precursor of NO, on energy metabolism by BAT and skeletal muscle, as well as glucose metabolism in South Asian men compared with men of European descent.

**Methods:**

We included ten Dutch South Asian men (age 46.5 ± 2.8 years, BMI 30.1 ± 1.1 kg/m^2^) and ten Dutch men of European descent, that were similar with respect to age and BMI, with prediabetes (fasting plasma glucose level 5.6–6.9 mmol/l or plasma glucose levels 2 h after an OGTT 7.8–11.1 mmol/l). Participants took either l-arginine (9 g/day) or placebo orally for 6 weeks in a randomised double-blind crossover study. Participants were eligible to participate in the study when they were aged between 40 and 55 years, had a BMI between 25 and 35 kg/m^2^ and did not have type 2 diabetes. Furthermore, ethnicity was defined as having four grandparents of South Asian or white European origin, respectively. Blinding of treatment was done by the pharmacy (Hankintatukku) and an independent researcher from Leiden University Medical Center randomly assigned treatments by providing a coded list. All people involved in the study as well as participants were blinded to group assignment. After each intervention, glucose tolerance was determined by OGTT and basal metabolic rate (BMR) was determined by indirect calorimetry; BAT activity was assessed by cold-induced [^18^F]fluorodeoxyglucose ([^18^F]FDG) positron emission tomography–computed tomography scanning. In addition, a fasting skeletal muscle biopsy was taken and analysed ex vivo for respiratory capacity using a multisubstrate protocol. The primary study endpoint was the effect of l-arginine on BAT volume and activity.

**Results:**

l-Arginine did not affect BMR, [^18^F]FDG uptake by BAT or skeletal muscle respiration in either ethnicity. During OGTT, l-arginine lowered plasma glucose concentrations (AUC_0–2 h_ − 9%, *p* < 0.01), insulin excursion (AUC_0–2 h_ − 26%, *p* < 0.05) and peak insulin concentrations (−26%, *p* < 0.05) in Europid but not South Asian men. This coincided with enhanced cold-induced glucose oxidation (+44%, *p* < 0.05) in Europids only. Of note, in skeletal muscle biopsies several respiration states were consistently lower in South Asian men compared with Europid men.

**Conclusions/interpretation:**

l-Arginine supplementation does not affect BMR, [^18^F]FDG uptake by BAT, or skeletal muscle mitochondrial respiration in Europid and South Asian overweight and prediabetic men. However, l-arginine improves glucose tolerance in Europids but not in South Asians. Furthermore, South Asian men have lower skeletal muscle oxidative capacity than men of European descent.

**Funding:**

This study was funded by the EU FP7 project DIABAT, the Netherlands Organization for Scientific Research, the Dutch Diabetes Research Foundation and the Dutch Heart Foundation.

**Trial registration:**

ClinicalTrials.gov NCT02291458.

**Electronic supplementary material:**

The online version of this article (10.1007/s00125-018-4752-6) contains peer-reviewed but unedited supplementary material, which is available to authorised users.

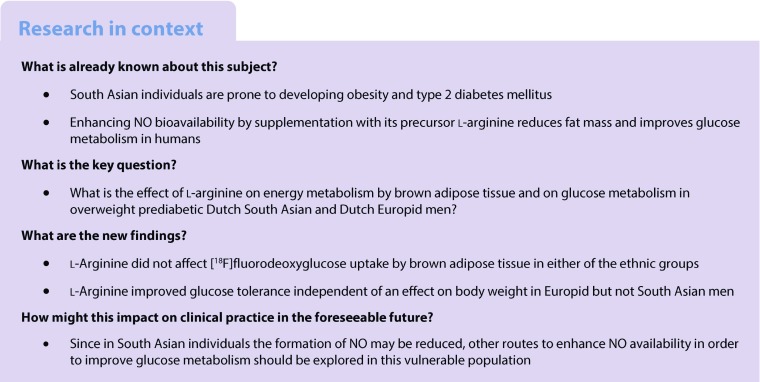



## Introduction

The South Asian population, comprising 20% of the total world population, is particularly vulnerable to developing obesity and type 2 diabetes mellitus. Currently, South Asians living in the Netherlands have a sixfold higher risk of developing type 2 diabetes compared with native Dutch Europids and are at higher risk of developing diabetes-related complications, including cardiovascular disease [1–4]. An important contributor is their highly common disadvantageous metabolic phenotype, consisting of central obesity, insulin resistance and dyslipidaemia [5]. This disadvantageous phenotype likely results from extrinsic factors such as migration followed by adaptation of a western lifestyle and less physical activity as well as intrinsic factors such as a disturbed energy metabolism (e.g. a reduced [fat] oxidative capacity) [6]. The current epidemic of type 2 diabetes results in high morbidity and mortality and novel treatment options to combat ‘diabesity’ are urgently needed. Since reduction of energy intake has been proven to be often ineffective in the long term due to poor compliance [7], shifting energy balance towards higher energy expenditure is an attractive therapeutic strategy.

Skeletal muscle mitochondrial function plays a pronounced role in whole-body energy expenditure and is closely associated with insulin sensitivity [8]. Poor skeletal muscle oxidative capacity may play a crucial role in the development of obesity-induced peripheral insulin resistance (and subsequently type 2 diabetes), so enhancing skeletal muscle mitochondrial function may improve whole-body glucose metabolism. Skeletal muscle and possibly also brown adipose tissue (BAT) are known to be involved in human energy metabolism. Due to the presence of uncoupling protein-1 (UCP-1) in the inner membrane of its mitochondria, BAT is able to combust relatively large amounts of fatty acids and glucose, resulting in dissipation of energy as heat rather than the production of ATP. Interestingly, a reduction in BAT volume and activity has been associated with both adiposity [9] and diabetic status [10] and we recently showed reduced BAT volume in young South Asian men compared with young Europid men [11]. Thus, enhancing BAT volume and activity could be a potential therapeutic approach to ameliorate type 2 diabetes risk, especially in South Asians. So far, significant BAT recruitment in humans has only been shown by means of prolonged intermittent cold exposure (i.e. cold acclimation) [12–14] or massive weight loss [15]. Other means to recruit BAT are urgently awaited.

Animal studies have suggested that nitric oxide (NO) plays a pivotal role in regulating mitochondrial biogenesis in skeletal muscle and BAT [16]; mice that lack the endothelial enzyme NO synthase (NOS), which catalyses the conversion of l-arginine to NO, display lower BAT volume, fewer mitochondria in BAT and muscle, lower energy expenditure and aggravated insulin resistance [17]. In addition, enhancing NO bioavailability by supplementation of its precursor l-arginine improves glucose metabolism and reduces fat mass in both animals and humans [18, 19], possibly due to effects on skeletal muscle respiration and BAT activity leading to enhanced energy expenditure [20–22]. Interestingly, South Asian individuals exhibit lower flow-mediated vasodilatation compared with Europids [23], pointing towards lower NO bioavailability. Thus, increasing NO bioavailability might be a promising approach to enhance skeletal muscle mitochondrial function as well as BAT activity, thereby exerting positive effects on whole-body energy and substrate metabolism.

The primary aim of the current study was to investigate the effects of 6 weeks of l-arginine supplementation on energy metabolism by BAT in overweight prediabetic Dutch South Asian and Dutch Europid men. In addition, we assessed the effects of l-arginine on skeletal muscle metabolism as well as glucose metabolism.

## Methods

For more details of methods, see electronic supplementary materials (ESM) Methods.

### Participants

Ten prediabetic overweight (BMI 25–35 kg/m^2^) Dutch South Asian males (age 40–55 years) and ten prediabetic Dutch Europid males that were similar with respect to age and BMI were included in the study. Prediabetes was defined as either fasting plasma glucose levels between 5.6 and 6.9 mmol/l or plasma glucose levels 2 h after an OGTT between 7.8 and 11.1 mmol/l [24]. Exclusion criteria included uncontrolled hypertension, hyper- or hypothyroidism, liver or kidney dysfunction, rigorous exercise, smoking and use of beta-blockers.

### Study approval

The study was approved by the Ethics Committee of Maastricht University Medical Center and all participants provided written informed consent. Procedures were conducted according to the principles of the Declaration of Helsinki.

### Study design

Participants ingested either l-arginine (9 g/day l-arginine divided over three servings; Argimax; Hankintatukku, Karkkila, Finland) or visually identical placebo tablets (Hankintatukku) for 6 weeks in a randomised double-blind crossover design, with a 4 week washout period in between. Each intervention period was followed by two consecutive experimental days. On the first day, an individualised cooling protocol was performed [12, 25]; blood samples were taken at thermoneutrality and after 2 h of cold exposure. Subsequently, an [^18^F]fluorodeoxyglucose ([^18^F]FDG) positron emission tomography–computed tomography (PET-CT) scan (Gemini TF PET-CT; Philips Healthcare, Best, the Netherlands) was performed for quantification of BAT volume and activity [12]. On the second day, a fasting skeletal muscle biopsy was taken from the vastus lateralis muscle. The freshly obtained skeletal muscle tissue was analysed ex vivo for respiratory capacity, using a multisubstrate protocol [26, 27], in essence according to Hoeks et al [28]. The remainder of the muscle biopsy was frozen for determination of protein levels by western blotting [29, 30]. At least an hour after the muscle biopsy, an OGTT was performed and body composition was determined by means of dual x-ray absorptiometry (Discovery A; Hologic, Bedford, MA, USA). Participants were instructed to refrain from heavy physical exercise for 48 h before the first experimental day, and standardised evening meals were prescribed the day before each experimental day.

Two Europid participants did not complete the study. One participant dropped out due to abdominal complaints during the first supplementation period (after de-blinding upon consultation with an independent physician, this appeared to be due to l-arginine) and one dropped out because he moved abroad. Both were replaced and baseline characteristics are based on calculations only including the two new participants.

### Plasma measurements

Plasma glucose, NEFA and triacylglycerol concentrations were determined with an automated spectrophotometer (ABX Pentra 400 autoanalyser, HORIBA Medical, Montpellier, France) by using enzymatic colorimetric kits. Plasma glycerol concentrations were measured with an enzymatic assay (Enzytec Glycerol; Roche Biopharm, Darmstadt, Germany) automated on a Cobas Fara spectrophotometric autoanalyser (Roche Diagnostics, Almere, the Netherlands). Insulin levels were analysed by using commercially available radioimmunoassay kits (Human Insulin–specific Radioimmunoassay; Millipore Corporation, Burlington, MA, USA).

### Calculations and statistical analyses

For the OGTT, AUC values were determined using the trapezoid rule [31]. Incremental values were calculated by deducting the area below the baseline value from total AUCs. Insulin sensitivity was estimated using the Matsuda index [32]. The insulinogenic index (IGI; Δ*I*_*0–30*_*/*Δ*G*_*0–30*_, where I is insulin and G is glucose) was used as a measurement of early insulin secretion [33]. The oral disposition index (DI_o_; [Δ*I*_*0–30*_*/*Δ*G*_*0–30*_]/fasting insulin) was used to estimate beta cell function relative to the prevailing level of insulin resistance [34]. Mixed model analysis with treatment, ethnicity and baseline glucose levels as fixed effects and subject-specific intercepts as random effects was used to assess the effect of l-arginine on glucose and insulin excursions after adjustment for baseline glucose levels.

Statistical analyses were performed with PASW Statistics 22.0 for Mac (IBM, Armonk, NY, USA). For normally distributed data, two-sided independent sample *t* tests were used to compare finding between ethnicities, and two-sided paired sample *t* tests were used to compare findings between placebo and l-arginine treatments. For not normally distributed data, Mann–Whitney *U* test and Wilcoxon signed rank test were used. ANCOVA was used to correct variables for fat-free mass. Pearson correlations were used to identify correlations between variables. Differences were considered to be statistically significant when *p* < 0.05. Data are reported as mean ± SEM.

## Results

### Participant characteristics and compliance

Twenty overweight Dutch South Asian and Europid men with prediabetes participated in this study. Characteristics of the participants are summarised in Table 1. At the start of the intervention, South Asian and Europid participants were comparable with respect to age (46.5 ± 2.8 vs 47.5 ± 2.0 years) and BMI (30.1 ± 1.1 vs 30.7 ± 1.2 kg/m^2^). South Asians were shorter than Europids (1.76 ± 0.02 vs 1.81 ± 0.02 m), although the difference did not reach statistical significance (*p* = 0.06). Fasting plasma glucose levels were similar between ethnicities (5.6 ± 0.2 vs 5.7 ± 0.2 mmol/l). In addition, the number of participants with isolated impaired fasting glucose (IFG), isolated impaired glucose tolerance (IGT) and combined IFG and IGT was comparable between ethnicities: six with IFG, two with IGT and two with combined IFG and IGT in the South Asian group; six with IFG, one with IGT and three with combined IFG and IGT in the Europid group.

**Table 1:** Characteristics of participants

Baseline	Europid (*n* = 10)	South Asian (*n* = 10)	All (*n* = 20)
Age (years)	47.5 ± 2.0	46.5 ± 2.8	47.0 ± 2.1
Height (m)	1.81 ± 0.02	1.76 ± 0.02	1.78 ± 0.02
BMI (kg/m^2^)	30.7 ± 1.2	30.1 ± 1.1	30.4 ± 1.1
Glucose (mmol/l)	5.7 ± 0.2	5.6 ± 0.2	5.6 ± 0.2

Data are expressed as mean ± SEM of values on the day of screening

Compliance with treatment was confirmed by counting returned supplements (<5% of supplements were returned). Supplements were well tolerated and the reported side effects were generally mild (e.g. mild dyspepsia), except in one study participant who withdrew from the study due to more severe dyspepsia and who was subsequently replaced (see ESM Methods).

### l-Arginine does not affect body composition

Six weeks of l-arginine supplementation did not influence body weight, BMI, fat mass or lean mass in either of the ethnic groups (Table 2). Bone mineral density also remained unchanged. When analysing both groups together, body composition was not changed due to the intervention.

**Table 2:** Effect of l-arginine on body composition and plasma variables of Europid and South Asian men

Variable	Europid (*n* = 10)		South Asian (*n* = 10)		All (*n* = 20)	
	Placebo	l-Arginine	Placebo	l-Arginine	Placebo	l-Arginine
Body weight (kg)	99 ± 4	100 ± 4	93 ± 4	92 ± 4	96 ± 4	96 ± 4
BMI (kg/m^2^)	30.5 ± 1.3	30.6 ± 1.3	30.0 ± 1.1	29.9 ± 1.0	30.2 ± 1.2	30.3 ± 1.2
Fat mass (kg)	30.0 ± 2.0	31.6 ± 2.1	29.5 ± 2.4	29.6 ± 2.0	29.7 ± 2.1	30.6 ± 2.0
Fat mass (%)	30.1 ± 1.0	31.0 ± 1.1	31.2 ± 1.3	31.4 ± 1.1	30.7 ± 1.1	31.2 ± 1.1
Lean mass (kg)	65.8 ± 2.2	66.6 ± 2.4	61.1 ± 2.0	61.2 ± 2.1	63.3 ± 2.2	63.9 ± 2.4
Lean mass (%)	67.6 ± 0.9	67.0 ± 1.1	66.3 ± 1.3	66.5 ± 1.0	66.9 ± 1.1	66.7 ± 1.0
BMD (g/cm^3^)	1.26 ± 0.02	1.26 ± 0.03	1.25 ± 0.03	1.24 ± 0.03	1.25 ± 0.03	1.25 ± 0.03
Glucose (mmol/l)	5.6 ± 0.2	5.5 ± 0.1	5.3 ± 0.1	5.3 ± 0.1	5.5 ± 0.2	5.4 ± 0.1
Insulin (pmol/l)	160 ± 35	125 ± 21	125 ± 35	132 ± 21	139 ± 35	132 ± 21
Triacylglycerol (mmol/l)	1.5 ± 0.2	1.5 ± 0.2	1.6 ± 0.2	1.9 ± 0.4	1.6 ± 0.2	1.7 ± 0.3
NEFA (mmol/l)	0.49 ± 0.07	0.53 ± 0.07	0.59 ± 0.03	0.58 ± 0.04	0.54 ± 0.05	0.55 ± 0.05
Free glycerol (μmol/l)	77 ± 7	85 ± 6	90 ± 8	90 ± 9	84 ± 7	87 ± 7

Data are expressed as mean ± SEM

BMD, bone mineral density

### l-Arginine enhances cold-induced glucose oxidation in Europid men only

**Fig. 1:**
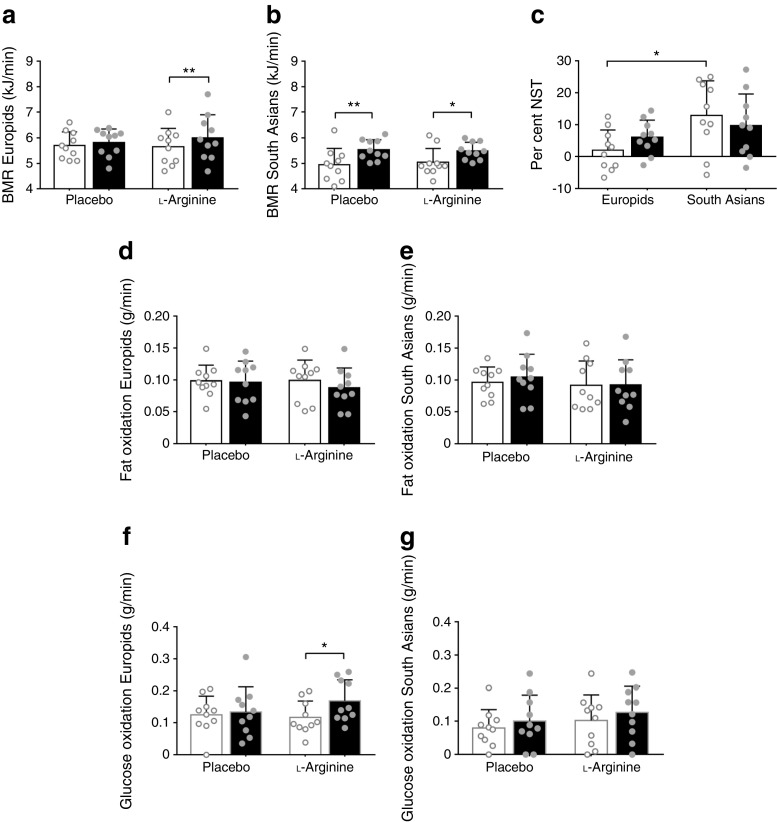
l-Arginine enhances cold-induced glucose oxidation in Europid men only. After supplementation with placebo and l-arginine, BMR (**a**, **b**), fat oxidation (**d**, **e**) and glucose oxidation (**f**, **g**) were assessed during thermoneutrality (white bars) and upon cold exposure (black bars) in Europid and South Asian men. Per cent NST (**c**) was calculated from the cold-induced increase in BMR after placebo (white bars) and l-arginine (black bars). Data are presented as mean ± SD. *p < 0.05 and **p < 0.01

We first examined whether energy expenditure was changed by 6 weeks of l-arginine supplementation. To this end, we measured energy expenditure in resting thermoneutral conditions (basal metabolic rate [BMR]) and under mild cold conditions (non-shivering thermogenesis [NST]). In accordance with our previous findings [11], BMR was significantly lower in South Asian individuals than in Europids after placebo (4.9 ± 0.2 vs 5.7 ± 0.2 kJ/min, *p* < 0.05). However, this difference disappeared after correction for fat-free mass with ANCOVA (ESM Fig. 1a, b; placebo, *p* value intercept = 0.082; l-arginine, *p* value intercept = 0.136). Furthermore, l-arginine did not influence BMR in either Europid or South Asian men (Fig. 1a, b). NST was significantly higher in South Asians (Fig. 1c).

Basal fat and glucose oxidation were similar in South Asians and Europids (Fig. 1d–g), as was the respiratory quotient (ESM Fig. 1c). Of note, l-arginine markedly enhanced cold-induced glucose oxidation in Europids only (+44%, *p* < 0.05, Fig. 1f, g). This was reflected by a higher RQ upon cold exposure after l-arginine treatment, although the difference was not statistically significant (ESM Fig. 1c).

### l-Arginine does not influence glucose uptake by BAT

**Fig. 2:**
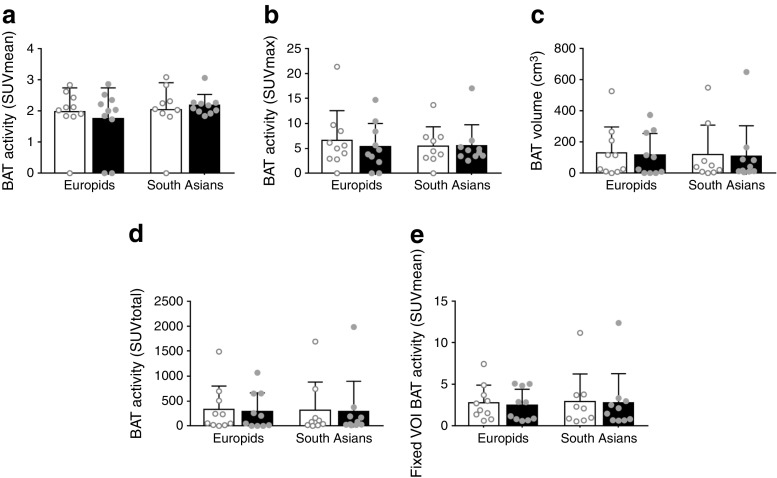
l-Arginine does not affect glucose uptake by BAT. We assessed mean (**a**) and maximal (**b**) cold-induced [^18^F]FDG uptake (expressed as SUV), BAT volume (**c**), total [^18^F]FDG uptake (SUVmax × BAT volume) (**d**) and [^18^F]FDG uptake by fixed volumes of interest (VOI) in BAT (**e**) in Europid and South Asian men after supplementation with placebo (white bars) or l-arginine (black bars). Data are presented as mean ± SD

We next assessed the effects of l-arginine on BAT metabolism by means of cold-induced [^18^F]FDG PET-CT scan. The mean and maximal BAT activity (expressed as the standardised uptake value [SUV]) did not differ between the ethnic groups and were not influenced by l-arginine treatment (Fig. 2a, b). In addition, detectable BAT volumes and total BAT activity were similar when comparing South Asians and Europids and were not influenced by l-arginine in either ethnicity (Fig. 2c, d). When analysing both ethnic groups together, l-arginine did not change BAT activity and volume (data not shown).

Because BAT activity could not be detected in all participants, we also used the fixed volume method [26] to specifically determine activity in predetermined volumes of interest in the supraclavicular adipose tissue depot. We found that BAT activity in fixed volumes of interest was similar between South Asians and Europids and was not influenced by l-arginine treatment (Fig. 2e). In addition, radiodensity of this supraclavicular BAT depot (expressed in Hounsfield units) was not significantly different between ethnicities or after l-arginine treatment (data not shown).

We also determined cold-induced [^18^F]FDG uptake (expressed as mean SUV) in skeletal muscle, subcutaneous and visceral white adipose tissue, liver and brain. Similar to uptake in BAT, [^18^F]FDG uptake in these tissues did not differ between South Asians and Europids and was not affected by l-arginine treatment (Table 3).

**Table 3:** Effect of l-arginine on [^18^F]FDG uptake (SUVmean) in skeletal muscle, subcutaneous and visceral WAT, liver and brain in Europid and South Asian men

SUVmean	Europid (*n* = 10)		South Asian (*n* = 10)		All (*n* = 20)	
	Placebo	l-Arginine	Placebo	l-Arginine	Placebo	l-Arginine
SM	0.63 ± 0.02	0.62 ± 0.02	0.61 ± 0.02	0.61 ± 0.02	0.62 ± 0.02	0.62 ± 0.02
sWAT	0.17 ± 0.01	0.17 ± 0.01	0.20 ± 0.01	0.19 ± 0.01	0.18 ± 0.01	0.18 ± 0.01
vWAT	0.31 ± 0.03	0.33 ± 0.02	0.29 ± 0.03	0.31 ± 0.03	0.30 ± 0.03	0.32 ± 0.02
Liver	2.43 ± 0.05	2.50 ± 0.08	2.42 ± 0.09	2.58 ± 0.09	2.43 ± 0.07	2.54 ± 0.09
Brain	8.8 ± 0.4	8.9 ± 0.5	8.3 ± 0.5	8.1 ± 0.4	8.5 ± 0.4	8.5 ± 0.5

Data are expressed as mean ± SEM

SM, skeletal muscle; sWAT, subcutaneous white adipose tissue; vWAT, visceral white adipose tissue

### l-Arginine improves glucose tolerance in Europid men only

**Fig. 3:**
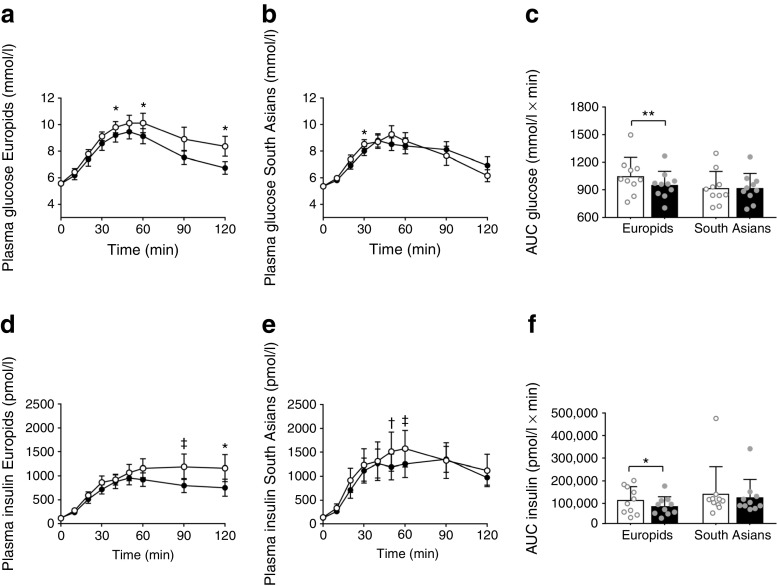
l-Arginine improves glucose tolerance in Europid men only. After placebo (white circles/bars) and l-arginine supplementation (black circles/bars), up to 120 min after ingestion of 75 g of dextrose, plasma glucose (**a**, **b**) and insulin excursions (**d**, **e**) were assessed in Europid and South Asian men. The AUC was calculated using the trapezoidal rule for glucose (**c**) and insulin (**f**). Data are presented as mean ± SEM (**a**, **b**, **d**, **e**) or as mean ± SD (**c**, **f**). *p < 0.05 and **p < 0.01 for placebo vs l-arginine; ^†^*p* = 0.07 and ^‡^*p* = 0.08 for placebo vs l-arginine

Since long-term treatment with l-arginine has previously been shown to improve glucose metabolism [35], we subsequently studied the effects of 6 weeks of l-arginine supplementation on plasma glucose variables as well as on lipid metabolism. l-Arginine did not influence fasting plasma levels of glucose, insulin, triacylglycerols, NEFA or free glycerol in either South Asian or Europid men (Table 2). When data for both groups were pooled, these fasting plasma levels were not affected by l-arginine supplementation.

l-Arginine improved glucose tolerance in Europid men, as demonstrated by reduced plasma glucose excursion during OGTT (AUC −9%, *p* < 0.01, Fig. 3a, c) and lower incremental glucose (−25%, *p* < 0.05, Table 4), but not in South Asian men (Fig. 3b, c and Table 4). This coincided with lower insulin excursion (AUC −26%, *p* < 0.05, Fig. 3d–f) and incremental insulin (−28%, *p* < 0.05, Table 4) in Europids only. After correction for baseline glucose levels by mixed model analysis, the glucose excursion was still significantly lower in the Europid men (*p* = 0.005); the insulin excursion was also lower but the difference did not reach statistical significance (*p* = 0.056). Furthermore, l-arginine reduced peak insulin in Europids (−26%, *p* < 0.05; Table 4) and, albeit not significantly, increased the Matsuda index (+13%, *p* = 0.06). This points towards enhanced insulin sensitivity. Of note, although differences were not statistically significant, Europids had higher peak glucose levels (*p* = 0.10) and peak glucose time (*p* = 0.10) after receiving placebo, compared with South Asians, but had a lower Matsuda index (*p* = 0.004), the latter pointing to enhanced insulin sensitivity. Beta cell function in relation to the level of insulin sensitivity, as assessed by DI_o_, was not affected by l-arginine in either Europids or South Asians Table 4).

**Table 4:** Effect of l-arginine on variables derived from an OGTT

Variable	Europid (*n* = 10)		South Asian (*n* = 10)	
	Placebo	l-Arginine	Placebo	l-Arginine
Peak glucose (mmol/l)	10.7 ± 0.6	10.1 ± 0.5	9.4 ± 0.5	9.1 ± 0.5^†††^
Peak glucose time (min)	62.0 ± 9.2	51.0 ± 5.7	44.0 ± 4.0	52.0 ± 5.1^††^
AUC_0–120_ glucose (mmol/l × min)	1052 ± 64	955 ± 47**	922 ± 57	921 ± 51
AUC_0–120_ incremental glucose (mmol/l × min)	382 ± 56	288 ± 33*	281 ± 45	278 ± 40
Peak insulin (pmol/l)	1479 ± 242	1096 ± 151*	1683 ± 389	1508 ± 277
Peak insulin time (min)	73 ± 10	60 ± 8	71 ± 9	70 ± 7
AUC_0–120_ insulin (pmol/l × min)	114,240 ± 19,046	87,514 ± 13,109*	142,012 ± 37,842	126,609 ± 25,157
AUC_120_ incremental insulin (pmol/l × min)	100,223 ± 17,397	72,415 ± 11,008*	125,077 ± 34,058	111,052 ± 22,492
Matsuda index	2.27 ± 0.37	2.56 ± 0.41^‡^	2.13 ± 0.30^††^	2.22 ± 0.33
HOMA-IR	4.21 ± 0.62	4.56 ± 0.81	4.92 ± 1.25	4.49 ± 0.88
IGI (pmol/mmol)	232 ± 45	224 ± 40	350 ± 78	388 ± 76
DI_o_	1.9 ± 0.2	1.9 ± 0.4	2.8 ± 0.6	3.5 ± 0.8

Values are presented as mean ± SEM

*p < 0.05 and **p < 0.01, l-arginine vs placebo; ^††^*p* < 0.01 and ^†††^*p* < 0.001, Europid vs South Asian; ^‡^*p* = 0.06, l-arginine vs placebo

### South Asian men exhibit lower skeletal muscle oxidative capacity, which is not influenced by l-arginine

NO is known to be involved in regulating mitochondrial function [16]. Since skeletal muscle mitochondrial function is closely associated with peripheral insulin sensitivity [8], we next assessed the effect of l-arginine on skeletal muscle respiratory capacity in the South Asian and Europid men. However, l-arginine did not influence any of the skeletal muscle respiratory states analysed (ESM Fig. 2a–f). Of note, several respiration states were consistently lower in South Asian compared with Europid men. We also examined protein expression of oxidative phosphorylation (OXPHOS) complexes in skeletal muscle and found that all complexes were similar between ethnicities and were not affected by l-arginine (ESM Fig. 3).

## Discussion

In the current study, we show that l-arginine, the precursor of NO, does not affect energy expenditure, cold-induced BAT activity or skeletal muscle mitochondrial respiration in Europid and South Asian men with prediabetes. l-arginine improves glucose tolerance only in Europid men, possibly by improving peripheral insulin sensitivity.

Contrary to our hypothesis, l-arginine did not enhance either BAT volume or BAT activity as measured via [^18^F]FDG uptake. Preclinical studies in which the conversion of l-arginine to NO was diminished showed that this resulted in lower BAT volume as well as fewer mitochondria in BAT and muscle [16, 17]. Furthermore, a study in sheep showed that dietary supplementation with arginine enhanced BAT volume by 60% [36]. Although the current study suggests that l-arginine does not affect BAT, at least in humans, it cannot be excluded that l-arginine affects BAT oxidation since we used a tracer that only measures glucose uptake rather than oxidation. Therefore, future studies using alternative tracers, such as [^11^C]acetate [37] will be needed to assess the effect of l-arginine on oxidative metabolism in BAT.

Furthermore, we previously showed that healthy lean South Asian men exhibit reduced BMR, even after correction for fat-free mass, and reduced BAT volume compared with Europid men [11]. However, in the current study, the difference in BMR disappeared after correction for fat-free mass. Curiously, NST was significantly higher in South Asians, while both BAT activity and detectable BAT volume were equal when comparing South Asian and Europid men. This is in line with findings of a previous study by Admiraal et al [38] but contradicts our previous notion that BAT volume and activity are lower in South Asian men, at least as measured via [^18^F]FDG uptake.

Furthermore, in our study, we did not find that l-arginine had any effect on body composition, including fat mass, as had previously been reported after l-arginine supplementation in obese individuals for 21 days [18] and 12 weeks [39]. A plausible explanation is that our treatment period of 6 weeks might have been too short to induce a reduction in fat mass. The reduction in fat mass observed after 21 days in the study of Lucotti et al [18] might have been caused by the interaction between l-arginine supplementation and the hypoenergetic diet and exercise intervention.

Our finding that l-arginine treatment improves glucose metabolism in overweight and obese prediabetic Europid men is in line with previous studies. Lucotti et al [18] showed that in obese individuals with type 2 diabetes, 21 days of l-arginine supplementation in a dose comparable with ours (8.3 g/day), on top of a hypoenergetic diet and exercise intervention, lowered fasting and postprandial glucose levels compared with placebo treatment. Moreover, l-arginine further reduced insulin levels, suggesting improved insulin sensitivity. Indeed, supplementation with l-arginine for 18 months improved insulin sensitivity [35]. In our study, the lower glucose excursion during OGTT upon l-arginine treatment in Europids, combined with the lower insulin excursion and tendency towards increased Matsuda index, points to improved insulin sensitivity. By quantifying [^18^F]FDG uptake, we could not identify the metabolic organ that was most responsible for this beneficial metabolic effect. Still, several other mechanisms are plausible when seeking to explain the improved glucose metabolism. For instance, due to NO-induced vasodilatation, l-arginine can increase blood flow and thereby glucose supply to metabolic tissues [40]. Indeed, in individuals with type 2 diabetes, a 3 month intervention with l-arginine decreased vascular resistance and increased blood flow, further supporting this hypothesis [41].

An interesting result in the current study is the lack of effect of l-arginine on glucose metabolism in men of South Asian ethnicity. Since, to our knowledge, this is the first study in which individuals of South Asian descent have been treated with l-arginine, we can only speculate on possible underlying mechanisms. A recent study has shown that endothelial cells isolated from South Asian men display lower expression levels of endothelial NOS, one of the enzymes that converts l-arginine into NO [42], compared with cells isolated from a matched control group of European origin [43]. A reduced functionality of endothelial NOS in South Asians may result in lower NO formation and thus less-beneficial NO-mediated effects on organ perfusion and glucose metabolism. Thus, in South Asians, other routes to enhance NO availability should be explored.

We also observed a lower skeletal muscle oxidative capacity in South Asian men in the current study. Skeletal muscle mitochondrial oxidative capacity has been repeatedly linked to metabolic dysfunction, such as insulin resistance, although the causal relationships are less clear [44]. Recently, it has been reported that lean African-American women, who showed lower peripheral insulin sensitivity compared with matched white women, also showed lower skeletal muscle mitochondrial respiration [45], indicating a role for ethnicity in these impairments. Thus, ethnically inherited defects in mitochondrial capacity may render South Asians more prone to the development of disturbances in skeletal muscle energy metabolism and insulin resistance.

This study is not without limitations. At baseline, South Asian and Europid men were not fully comparable with respect to several metabolic variables. Participants were equal with respect to BMI but South Asians generally have a different body composition with more fat mass and less lean mass compared with Europids [11]. In the current study, however, fat mass did not significantly differ between ethnicities. Furthermore, at baseline, Europid men were more insulin sensitive, as was evident from a lower Matsuda index after placebo treatment. Europid men also showed signs of greater beta cell failure compared with South Asians, as apparent from the OGTT data. It can also be questioned whether beta cell mas was equal between both ethnicities at baseline. One of the effects of l-arginine is stimulation of insulin release by beta cells and this could have influenced the response to l-arginine in both ethnicities. However, if that was the case, one would have expected a lower rather than a higher response in the Europid group. Furthermore, BMR was lower in South Asian men, although this difference disappeared after correction for free-fat mass. L-Arginine may also have influenced appetite. Although participants consumed a standardised meal the evening before the study, we cannot exclude the possibility that longer-term differences in food intake or food preference may have influenced the metabolic outcomes of the current study. Unfortunately, we did not record objective data on appetite following l-arginine supplementation.

In conclusion, we show that 6 weeks of l-arginine supplementation does not affect BMR, [^18^F]FDG uptake by BAT or skeletal muscle mitochondrial respiration in Europid and South Asian overweight and obese prediabetic men. However, l-arginine improves glucose tolerance in Europid men but not in South Asian men. Furthermore, we show for the first time that South Asian men have lower skeletal muscle respiratory capacity compared with Europid men.

## Electronic supplementary material

ESM (PDF 1.57 MB)

## Abbreviations

BAT: Brown adipose tissue
BMR: Basal metabolic rate
DI_o_: Oral disposition index
[^18^F]FDG: [^18^F]fluorodeoxyglucose
IFG: Impaired fasting glucose
IGI: Insulinogenic index
IGT: Impaired glucose tolerance
NOS: NO synthase
NST: Non-shivering thermogenesis
PET-CT: Positron emission tomography–computed tomography
SUV: Standardised uptake value
