# Supplementary material for: Effect of l-arginine on energy metabolism, skeletal muscle and brown adipose tissue in South Asian and Europid prediabetic men: a randomised double-blinded crossover study
Source: Diabetologia. 2018 Oct 30;62(1):112–22. doi: 10.1007/s00125-018-4752-6 (PMC6290676; doi:10.1007/s00125-018-4752-6)
Supplement: Supplementary file 1 — (PDF 1.57 MB) [file 125_2018_4752_MOESM1_ESM.pdf]

Supplementary Materials and Methods

Participants

Ten pre-diabetic overweight (body mass index (BMI), 25-35 kg/m^2) Dutch South Asian males (age 40-55 years) and ten age- and BMI-matched pre-diabetic Dutch Europid males were included in the study. Ethnicity was defined as having four grandparents of South Asian or Europid origin, respectively. Subjects underwent a medical screening including their medical history, a physical examination, blood biochemistry tests, and an oral glucose tolerance test (OGTT) to exclude individuals with undiagnosed type 2 diabetes according to the 2014 American Diabetes Association (ADA) criteria (1). Pre-diabetes was defined as having either fasting plasma glucose levels between 5.6 and 6.9 mmol/l or plasma glucose levels 2h after an OGTT between 7.8 and 11.1 mmol/l (1). Exclusion criteria included uncontrolled hypertension, hyper- or hypothyroidism, liver or kidney dysfunction, rigorous exercise, smoking and use of beta-blockers. Three South Asians (2x ACE inhibitor, 1x angiotensin II receptor-blocker) and none of the Europids were taking antihypertensive medication before and during the intervention periods.

Study approval

The study was approved by the Ethics Committee of Maastricht University Medical Center and all participants provided written informed consent. Procedures were conducted according to the principles of the Declaration of Helsinki.

Study design

The study was conducted between November 2014 and October 2015 in Maastricht, the Netherlands. Subjects ingested either L-arginine (Argimax, Hankintatukku Oy, Karkkila, Finland) or placebo supplements (9 g/day) for six weeks in a randomized double-blind cross-over design, with a 4-week washout period in between. Blinding of treatment was done by Hankintatukku Oy and an independent researcher from the LUMC randomly assigned treatment to the subjects by providing a coded list. Supplements were divided over 3 gifts: after breakfast, lunch and dinner. Subjects were instructed to maintain their normal dietary habits during the 6-week intervention periods. Each intervention period was

followed by two experimental days and the last gift of L-arginine was taken the evening before the study days. During the first day an individualized cooling protocol followed by [^{18}F]fluorodeoxyglucose ([^{18}F]FDG) Positron Emission Tomography-Computed Tomography (PET-CT) scan (Gemini TF PET-CT, Philips Healthcare, Best, the Netherlands) for quantification of BAT volume and activity was performed (2). On the second day, a fasting skeletal muscle biopsy was taken from the vastus lateralis muscle. Furthermore, an oral glucose tolerance test (OGTT) was performed and body composition was determined by means of dual x-ray absorptiometry (DEXA, Discovery A, Hologic, Bedford, MA, USA). Subjects were instructed to refrain from heavy physical exercise, caffeine and alcohol intake 48h before the first experimental day, and standardized evening meals were prescribed the day before each experimental day.

Two Europid subjects did not complete the study. One subject due to abdominal complains during the first supplementation period (after deblinding upon consultation with an independent physician this appeared to be L-arginine) and one subject because he moved abroad. Both subjects were replaced and baseline characteristics are based on calculations excluding these subjects.

Individualized cooling and PET-CT scanning

The individualized cooling protocol (2; 3) on the first experimental day was initiated at noon, after a 4-h fasting period. For this purpose, a cannula was inserted in the right antecubital vein for blood sampling during thermoneutral and mild cold conditions and injection of the [^{18}F]FDG tracer. A pressure cuff (MTP, Medisana, Kerkrade, the Netherlands) was placed on the left arm for measurement of blood pressure and a chest strap (Polar T31, Polar, Kempele, Finland) was attached for measurement of heart rate. Energy expenditure was measured continuously via a face-mask connected to an indirect calorimeter (EZcal, IDEE, Maastricht, the Netherlands).

Subjects were wrapped in a water-perfused suit (ThermaWrap Universal 3166, MTRE Advanced Technologies, Yavne, Israel), and measurements started with a 45-minute thermoneutral period. Hereafter, subjects were gradually cooled until a temperature just above their shivering point (which took approximately 30 minutes), and measured for another 30 minutes at this mild cold temperature. Non-shivering thermogenesis (NST) was

calculated as the percentage increase in energy expenditure above basal metabolic rate (measured at thermoneutrality) at the temperature just above an individual's shivering temperature. Subsequently, 74 MBq of [^{18}F]FDG was injected intravenously, while subjects remained in the mild cold water-perfused suit. One hour after injection of [^{18}F]FDG, a PET-CT scan was performed. The PET-CT imaging protocol started with a low-dose CT scan (120 kV, 30mAs), immediately followed by a static PET scan (6 to 7 bed positions, 4 min per bed position) covering the range from the skull to the abdomen.

PET-CT analysis

PET-CT scans were analyzed using PMOD software (version 3.0, PMOD Technologies, Zurich, Switzerland) by both the researcher (MJWH) and an experienced nuclear medicine physician (BB). BAT activity was defined as [^{18}F]FDG uptake >1.5 standardized uptake value (SUV) in fat tissue (Hounsfield units [HU] between -10 and -180). SUV was calculated as: ([^{18}F]FDG uptake [kBq/ml])/(injected dose [kBq]/patient weight [g]). Regions of interest were semi-automatically outlined for determination of mean (SUVmean) and maximal (SUVmax) [^{18}F]FDG uptake in BAT locations. All upper body BAT depots were included in this analysis. Not all subjects showed pronounced BAT activity, defined with the SUV threshold of >1.5. Therefore, fixed volumes of interest (VOIs; 2.67 cm^3) were also carefully selected in the supraclavicular adipose tissue region (HU between -10 and -180) in the area with the highest [^{18}F]FDG uptake. VOIs were also placed in subcutaneous and visceral white adipose tissue (WAT), skeletal muscle (deltoid, erector spinae, biceps and triceps brachii muscles; average activity of these muscle groups is presented as average skeletal muscle [^{18}F]FDG uptake), liver and brain, as described previously (4). As such, we were able to compare [^{18}F]FDG uptake (calculated as SUVmean) between these tissues and between placebo and L-arginine interventions. Due to technical reasons, we could not perform a PET-CT scan in 1 of the South Asian subjects (after placebo treatment).

Ex vivo skeletal muscle respiration

The fasting skeletal muscle tissue, acquired at the second study day, was instantly placed in ice-cold preservation medium (BIOPS, OROBOROS Instruments, Innsbruck, Austria) and used for the preparation of permeabilized muscle fibers, as described previously (5).

Subsequently, oxygen consumption of these permeabilized muscle fibers (2.5-3.0 mg wet weight) was measured using an oxygraph (OROBOROS Instruments, Innsbruck, Austria), in essence according to Hoeks *et al.* (6). Respiration chambers were hyperoxygenated to ~360 μmol/l O_2. State 2 respiration was initiated by addition of malate (4 mM) and octanoylcarnitine (4 mM). Subsequently, ADP was added to evaluate coupled (state 3) respiration. Coupled respiration was then maximized by subsequent addition of glutamate (10 mM) and succinate (10 mM). Finally, the chemical uncoupler FCCP was titrated to evaluate the maximal capacity of the electron transport chain (State U). The integrity of the outer mitochondrial membrane was assessed by addition of cytochrome C (20 μM) upon maximal coupled respiration. Respiration measurements that displayed a cytochrome C response >10% above maximal coupled respiration were excluded from analysis. All measurements were performed in quadruplicate.

Muscle biopsy analysis

Protein levels were determined by Western blotting according to standard procedures. Primary antibodies directed against α-sarcomeric actin (loading control; A2172, Sigma, Zwijndrecht, the Netherlands) and total OXPHOS human Western blot antibody cocktail (ab110411; Abcam, Cambridge, U.K.) were detected with appropriate secondary antibodies conjugated with IRDye680 or IRDye800 and detected using the Odyssey Near Infrared System (LI-COR, Westburg, Leusden, the Netherlands).

Oral glucose tolerance test

To assess glucose tolerance in participants, an OGTT was performed one hour following the muscle biopsy. A cannula was inserted in the antecubital vein for blood sampling and the participant drank 75 gram of dextrose dissolved in 300 mL of tap water. Blood was drawn at t = 0, 10, 20, 30, 40, 50, 60, 90 and 120 min. Blood samples were cooled on ice and centrifuged. Next, plasma was obtained, snap-frozen in liquid nitrogen and stored at -80° C until further analysis.

Plasma measurements

Plasma glucose, NEFA and triglycerides were determined with an automated spectrophotometer (ABX Pentra 400 autoanalyzer) by using enzymatic colorimetric kits. Plasma glycerol concentrations were measured with an enzymatic assay (Enzytec Glycerol; Roche Biopharm) automated on a Cobas Fara spectrophotometric autoanalyzer (Roche Diagnostics). Insulin levels were analyzed by using commercially available radioimmunoassay kits (Human Insulin–specific Radioimmunoassay, Millipore Corporation).

Calculations and statistical analyses

During OGTT, AUC values were determined using the trapezoid rule (7). Incremental values were calculated by deducting the area below the baseline value from total AUCs. Insulin sensitivity was estimated using the Matsuda index (8). The insulinogenic index (IGI; $\Delta I_{0\text{-}30}/ \Delta G_{0\text{-}30}$) was used as a measurement of early insulin secretion (9). The oral disposition index (DI_0; ($\Delta I_{0\text{-}30}/ \Delta G_{0\text{-}30}$)/fasting insulin) was used to estimate ß cell function relative to the prevailing level of insulin resistance (10).

Statistical analyses were performed with PASW Statistics 22.0 for Mac (IBM, Armonk, NY, USA). Normal distribution of all relevant parameters was tested with Sharpiro-Wilk test, for all subjects combined and the separate study groups (South Asians and Europids). For normally distributed data, two-sided independent sample *t* tests were used to compare finding between the study groups, and two-sided paired sample *t* tests were used to compare findings between placebo and L-arginine treatments. For not normally distributed data, Mann-Whitney U tests and Wicoxon signed rank tests were used, respectively. ANCOVA was used to correct parameters for fat free mass. Pearson correlations were used to identify correlations between variables. P-values<0.05 were considered statistically significant. Data are reported as mean±SEM.

References

1. American Diabetes A: Standards of medical care in diabetes--2014 (2014). Diabetes care 37 Suppl 1:S14-80
2. van der Lans AA, Hoeks J, Brans B, et al (2013) Cold acclimation recruits human brown fat and increases nonshivering thermogenesis. J Clin Invest 123:3395-3403

3. Hanssen MJ, Wierts R, Hoeks J, et al (2015) Glucose uptake in human brown adipose tissue is impaired upon fasting-induced insulin resistance. Diabetologia 58:586-595
4. Vosselman MJ, Brans B, van der Lans AA, et al (2013) Brown adipose tissue activity after a high-calorie meal in humans. Am J Clin Nutr 98:57-64
5. Phielix E, Schrauwen-Hinderling VB, Mensink M, et al (2008) Lower intrinsic ADP-stimulated mitochondrial respiration underlies in vivo mitochondrial dysfunction in muscle of male type 2 diabetic patients. Diabetes 57:2943-2949
6. Hoeks J, van Herpen NA, Mensink M, et al (2010) Prolonged fasting identifies skeletal muscle mitochondrial dysfunction as consequence rather than cause of human insulin resistance. Diabetes 59:2117-2125
7. Matthews JN, Altman DG, Campbell MJ, Royston P (1990) Analysis of serial measurements in medical research. BMJ 300:230-235
8. Matsuda M, Defronzo RA (1999) Insulin sensitivity indices obtained from oral glucose tolerance testing: comparison with the euglycemic insulin clamp. Diabetes Care 22:1462-1470
9. Tura A, Kautzky-Willer A, Pacini G (2006) Insulinogenic indices from insulin and C-peptide: comparison of beta-cell function from OGTT and IVGTT. Diabetes Research and Clinical Practice 72:298-301
10. Retnakaran R, Qi Y, Goran MI, Hamilton JK (2009) Evaluation of proposed oral disposition index measures in relation to the actual disposition index. Diabetes Medicine 26:1198-1203

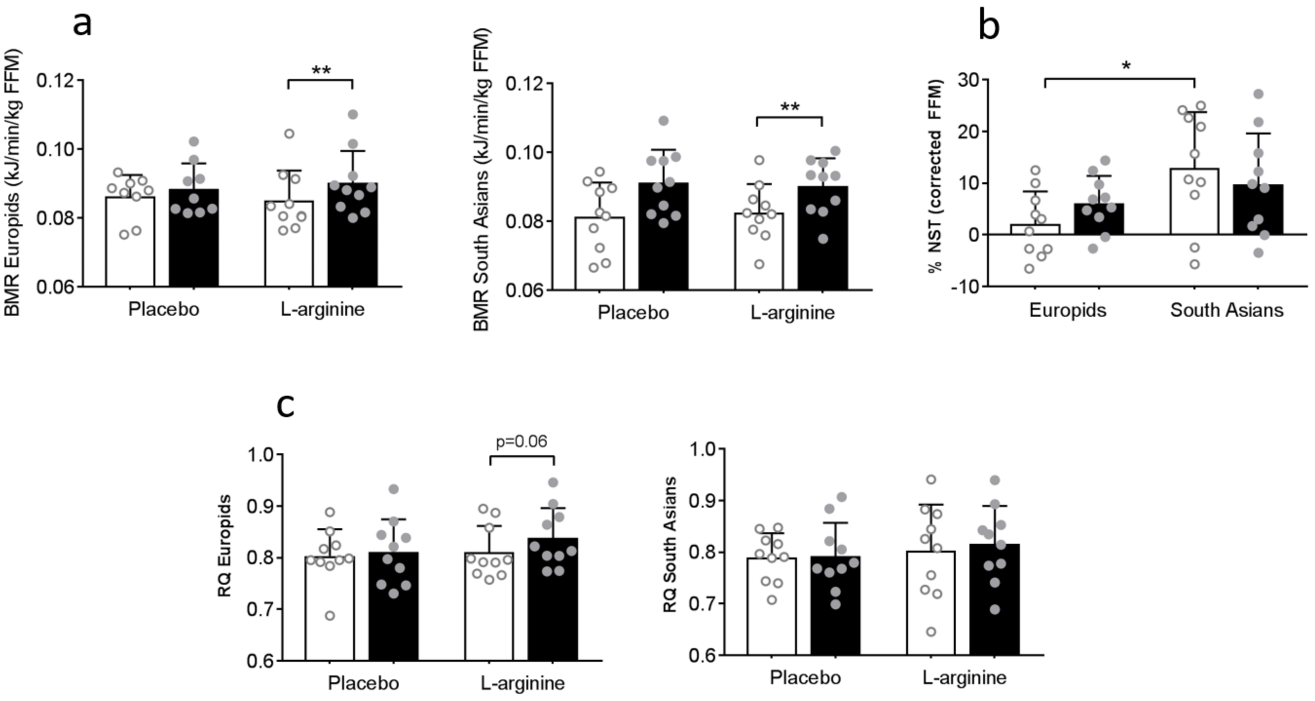

Suppl. Figure 1. Effects of L-arginine on energy expenditure corrected for fat mass and respiratory quotient. After supplementation with placebo (white bars) and L-arginine (black bars), basal metabolic rate (BMR) corrected for fat free mass (FFM) (a) and respiratory quotient (c) were assessed during thermoneutrality (TN) and upon cold exposure (COLD) in Europid and South Asian men. % non-shivering thermogenesis (NST) corrected for FFM (b) was calculated from the cold-induced increase in BMR. Data are presented as mean ± SEM. *P<0.05, **P<0.01.

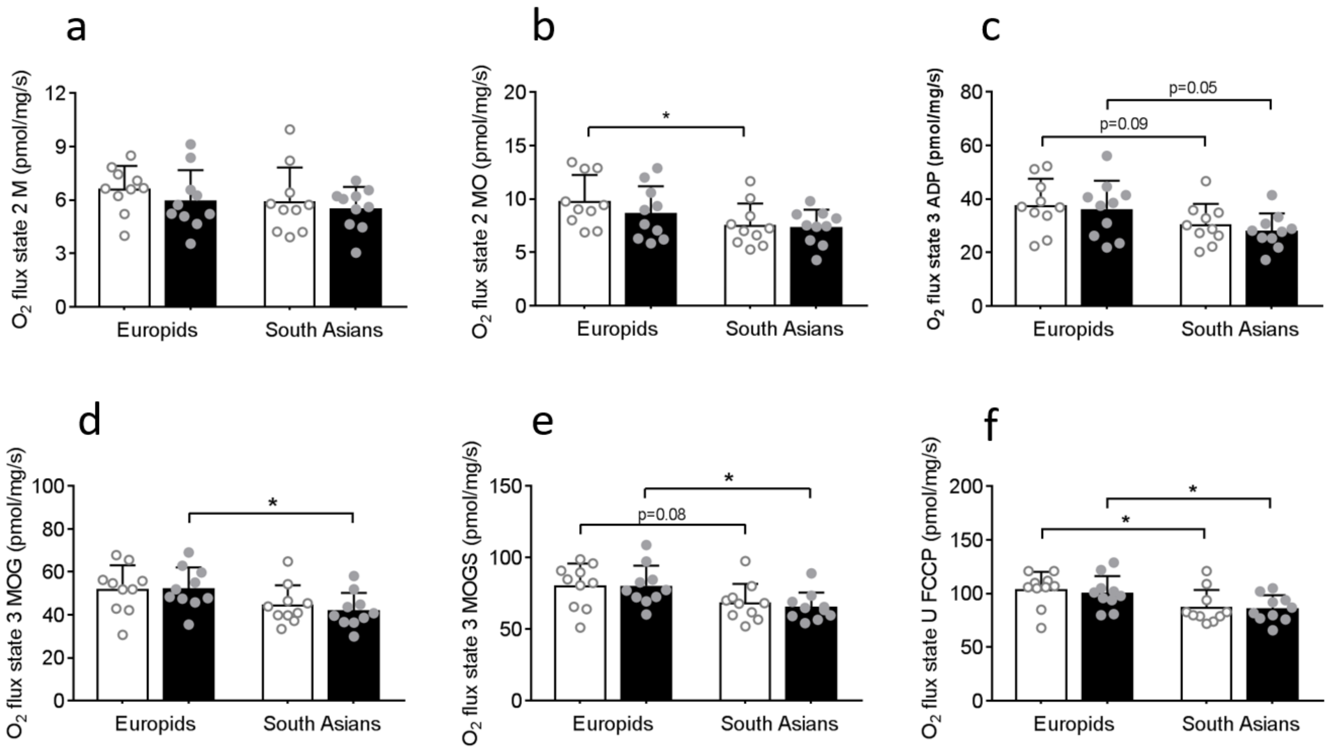

Suppl. Figure 2. South Asian men exhibit lower skeletal muscle oxidative capacity which is not affected by L-arginine. *Ex vivo* mitochondrial respiration was assessed in permeabilized vastus lateralis muscle fibers in Europid and South Asian men after placebo (white bars) and L-arginine (black bars) supplementation. Respiration upon addition of substrates only: malate (a) and malate + octanoyl carnitine (b); ADP-stimulated respiration upon addition of several mitochondrial complex I and complex II-linked substrates (state 3): malate + octanoyl carnitine (c), malate + octanoyl carnitine + glutamate (d), malate + octanoyl carnitine + glutamate + succinate (e). Maximally uncoupled respiration upon addition of the chemical uncoupler FCCP (state U) (f). Data are presented as mean ± SEM. *P<0.05. G, glutamate; M, malate; O, octanoyl carnitine; S, succinate.

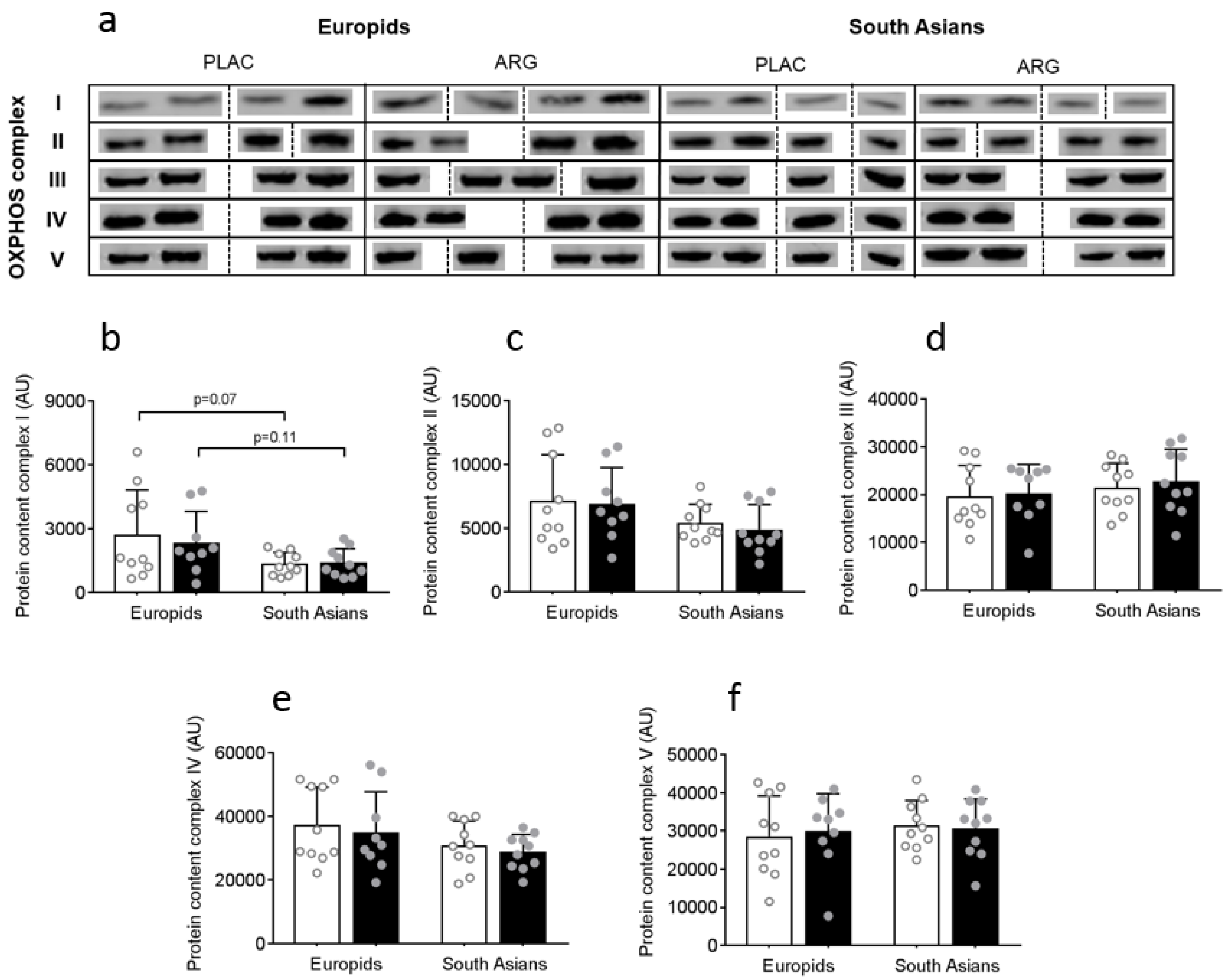

Suppl. Figure 3. Effects of L-arginine on OXPHOS protein content in skeletal muscle. After supplementation with placebo (white bars) and L-arginine (black bars), protein content of the individual complexes of the electron transport chain were quantified by Western blotting in vastus lateralis muscle of all subjects. An antibody cocktail that detects all five complexes was used (a), and the data are represented as mean ± SEM for the individual complexes (b-f). The original blots (g,i,k) with the chosen representative bands (h,j,l) are shown in the pages below.

g

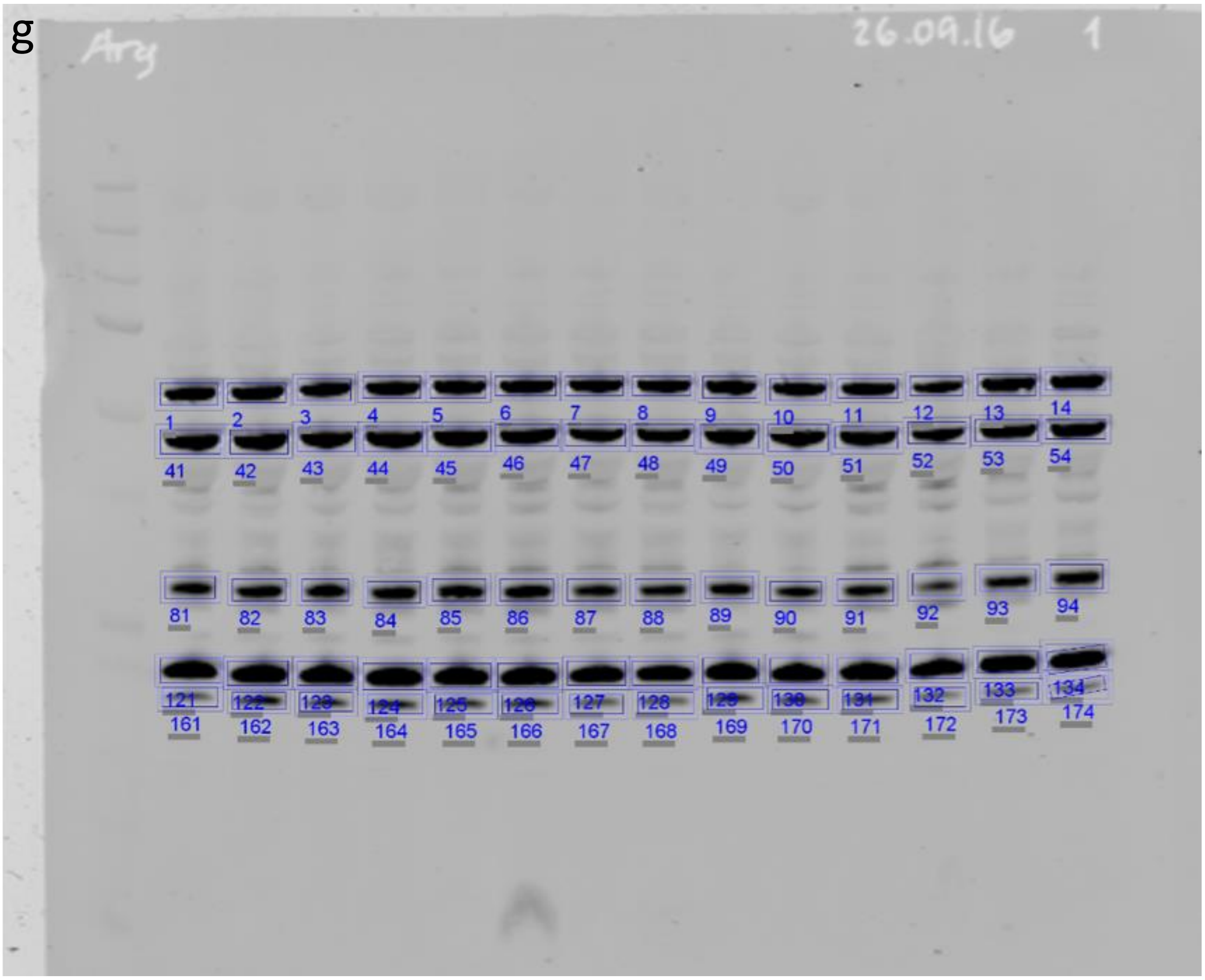

h

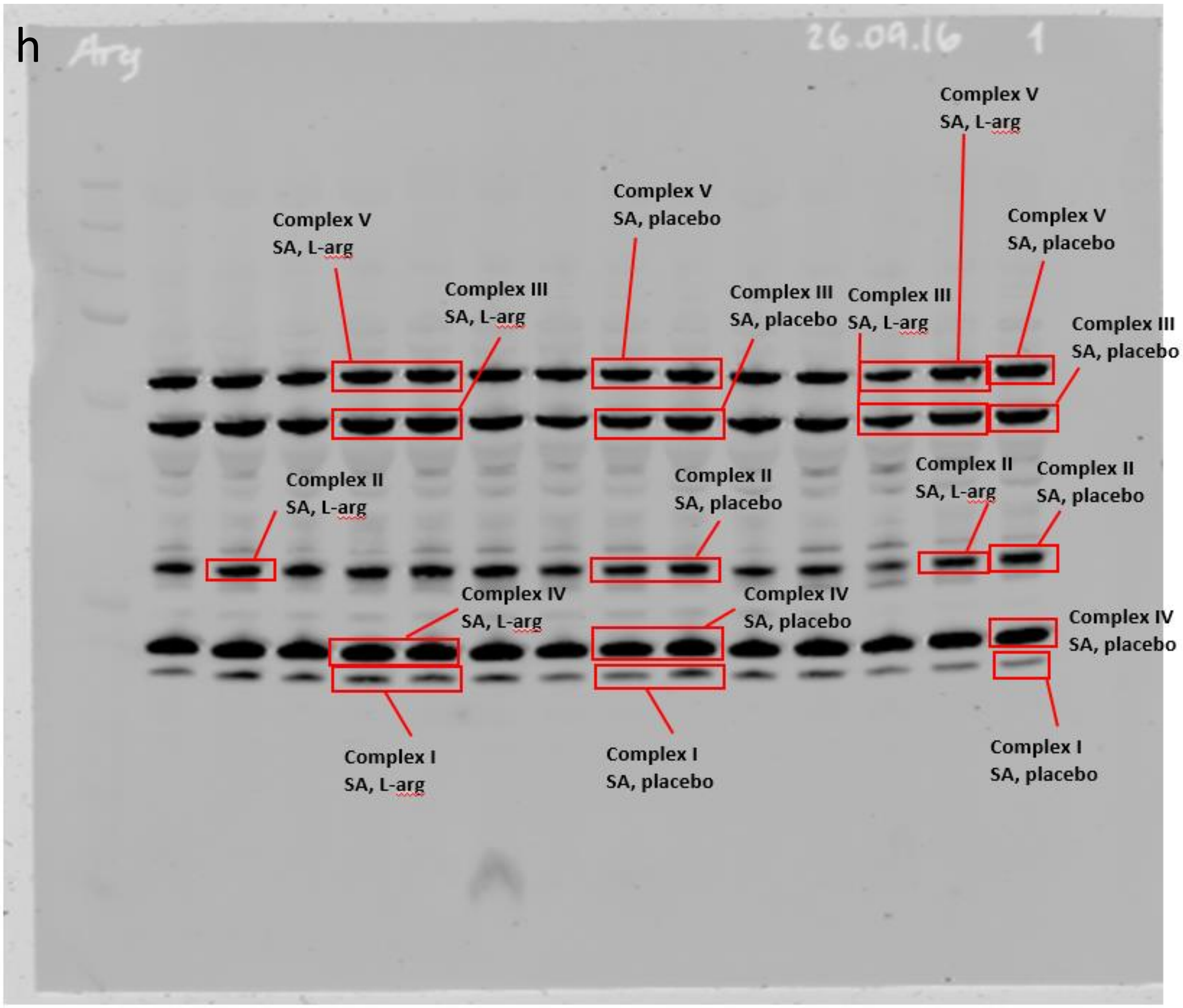
Arg
26.09.16 1
Complex V
SA, L-arg
Complex V
SA, placebo
Complex V
SA, L-arg
Complex V
SA, placebo
Complex III
SA, L-arg
Complex III
SA, placebo
Complex III
SA, L-arg
Complex III
SA, placebo
Complex II
SA, L-arg
Complex II
SA, placebo
Complex II
SA, L-arg
Complex II
SA, placebo
Complex IV
SA, L-arg
Complex IV
SA, placebo
Complex IV
SA, placebo
Complex I
SA, L-arg
Complex I
SA, placebo
Complex I
SA, placebo

i

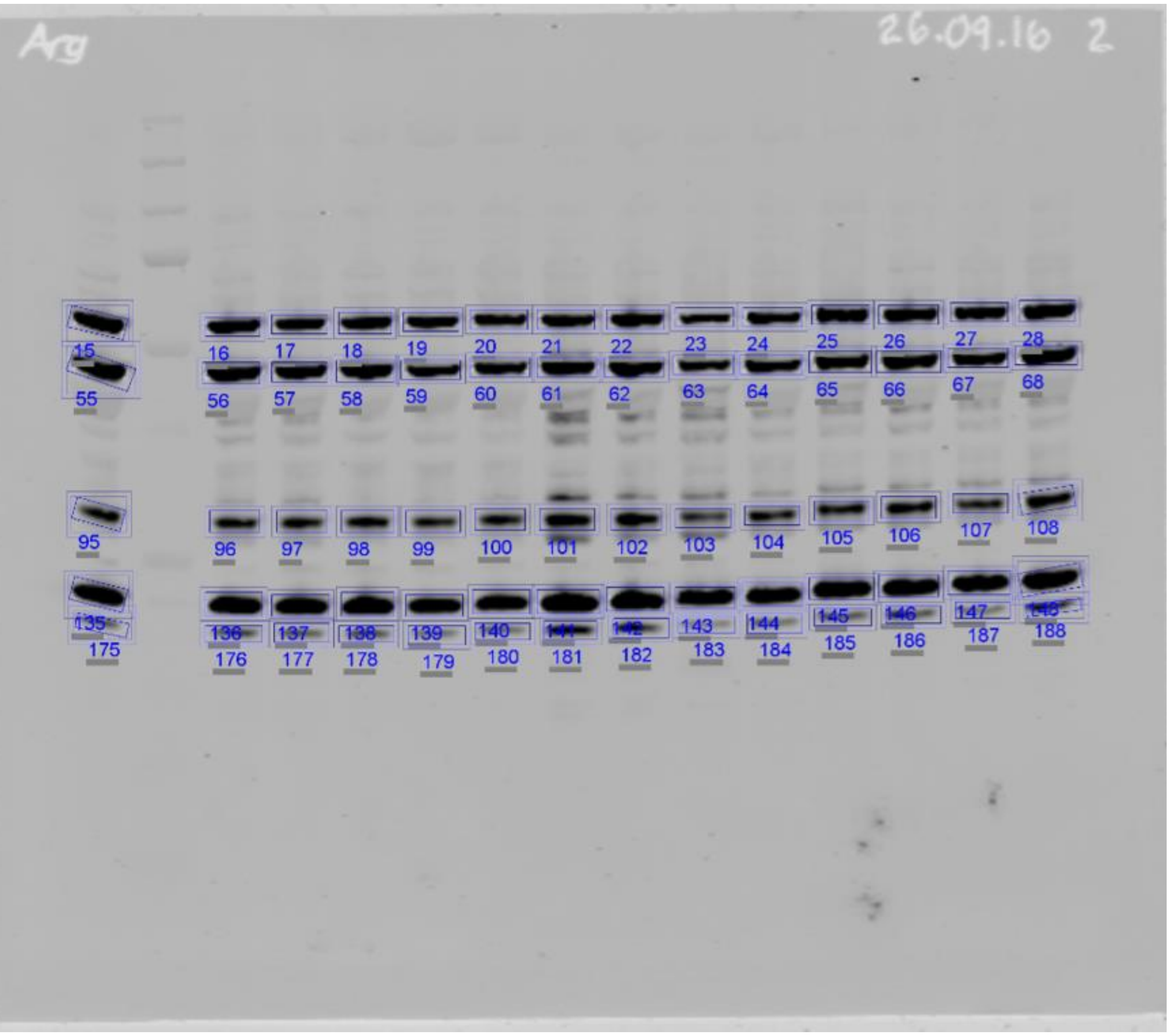
Arg
26.09.16 2
15 16 17 18 19 20 21 22 23 24 25 26 27 28
55 56 57 58 59 60 61 62 63 64 65 66 67 68
95 96 97 98 99 100 101 102 103 104 105 106 107 108
135 136 137 138 139 140 141 142 143 144 145 146 147 148
175 176 177 178 179 180 181 182 183 184 185 186 187 188

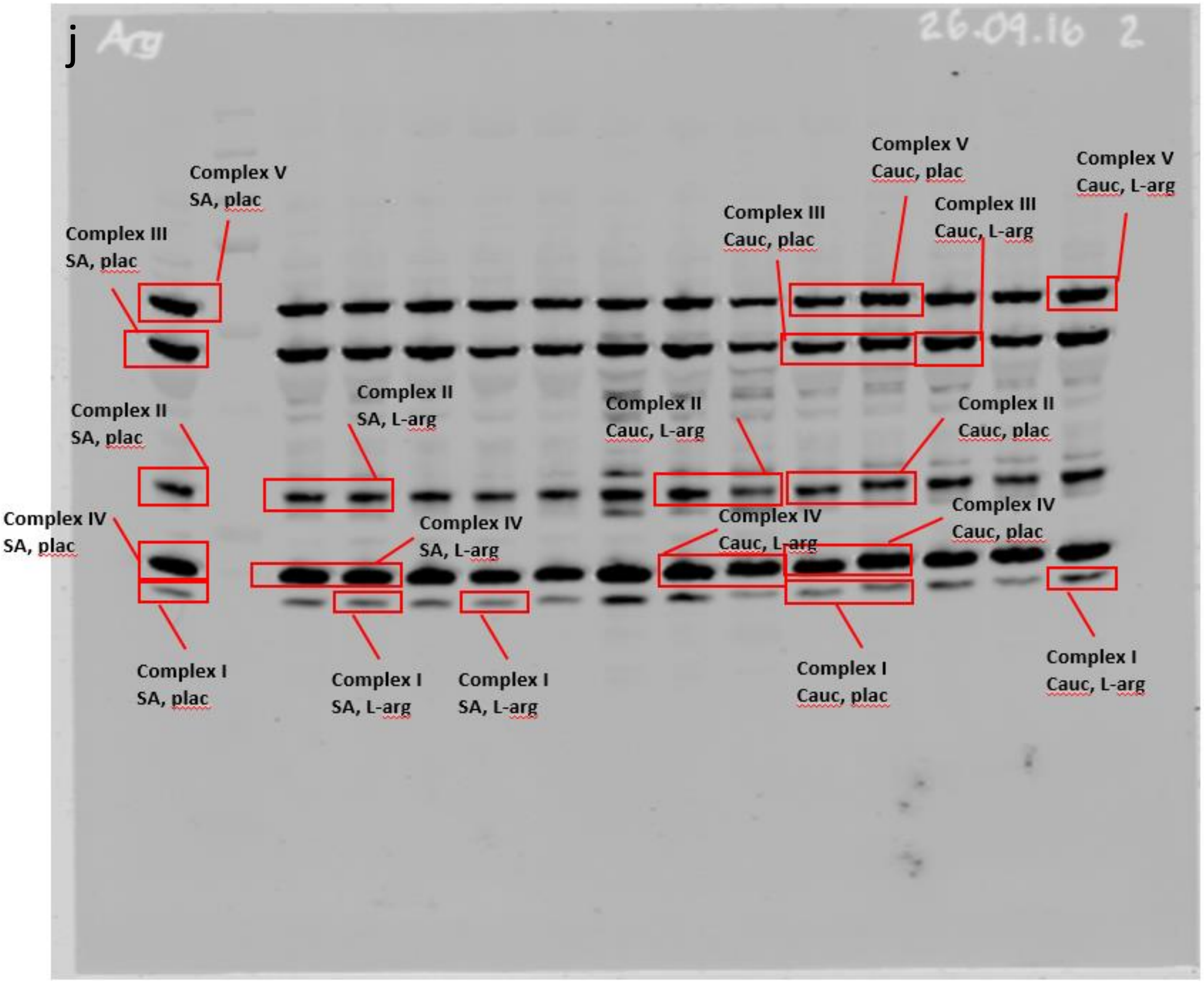
j
Arg
26.09.16 2
Complex V
SA, plac
Complex III
SA, plac
Complex II
SA, plac
Complex IV
SA, plac
Complex I
SA, plac
Complex II
SA, L-arg
Complex IV
SA, L-arg
Complex I
SA, L-arg
Complex I
SA, L-arg
Complex V
Cauc, plac
Complex III
Cauc, plac
Complex III
Cauc, L-arg
Complex V
Cauc, L-arg
Complex II
Cauc, L-arg
Complex II
Cauc, plac
Complex IV
Cauc, L-arg
Complex IV
Cauc, plac
Complex I
Cauc, plac
Complex I
Cauc, L-arg

k

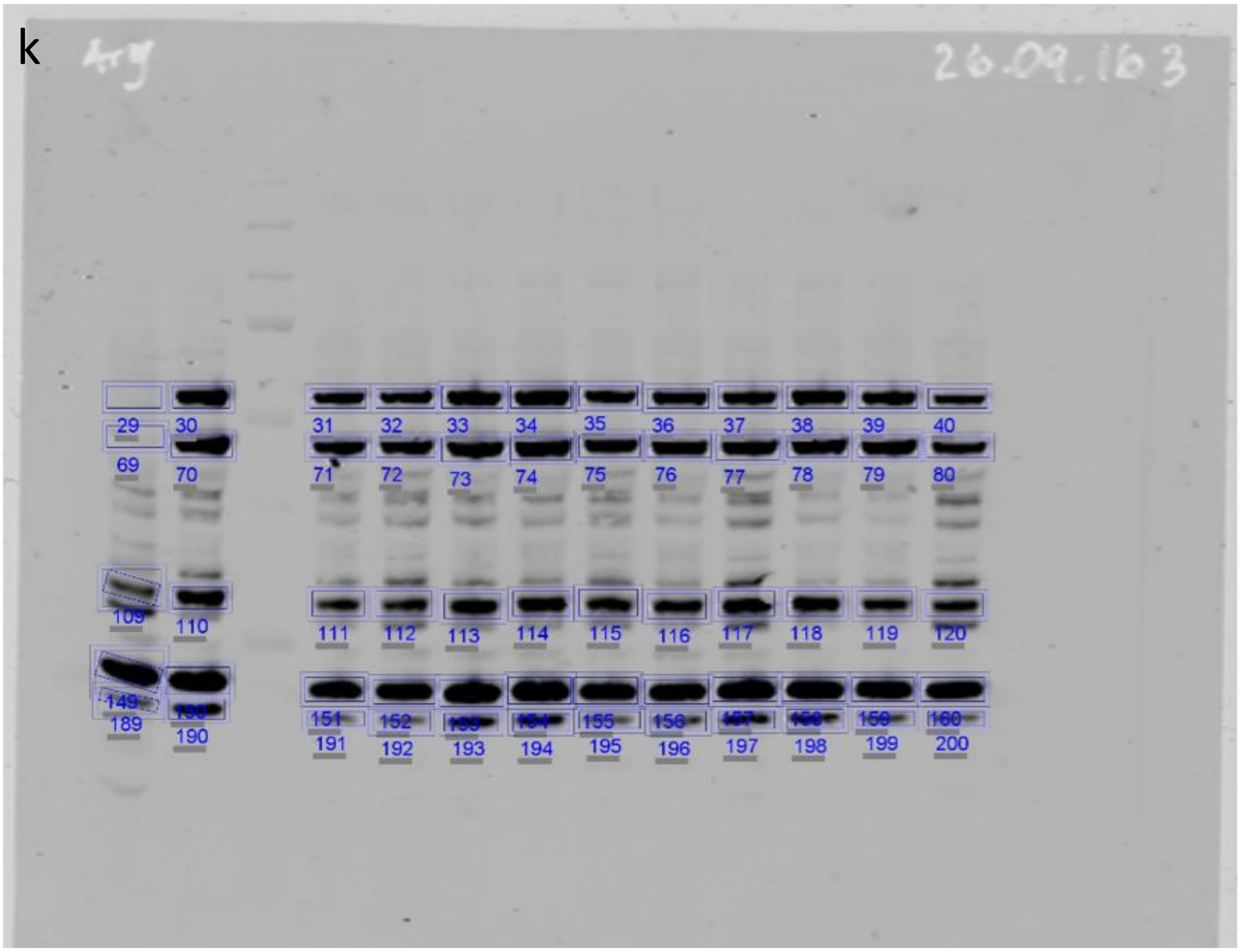

Arg
26.09.16 3
29
30
31
32
33
34
35
36
37
38
39
40
69
70
71
72
73
74
75
76
77
78
79
80
109
110
111
112
113
114
115
116
117
118
119
120
149
151
152
160
189
190
191
192
193
194
195
196
197
198
199
200

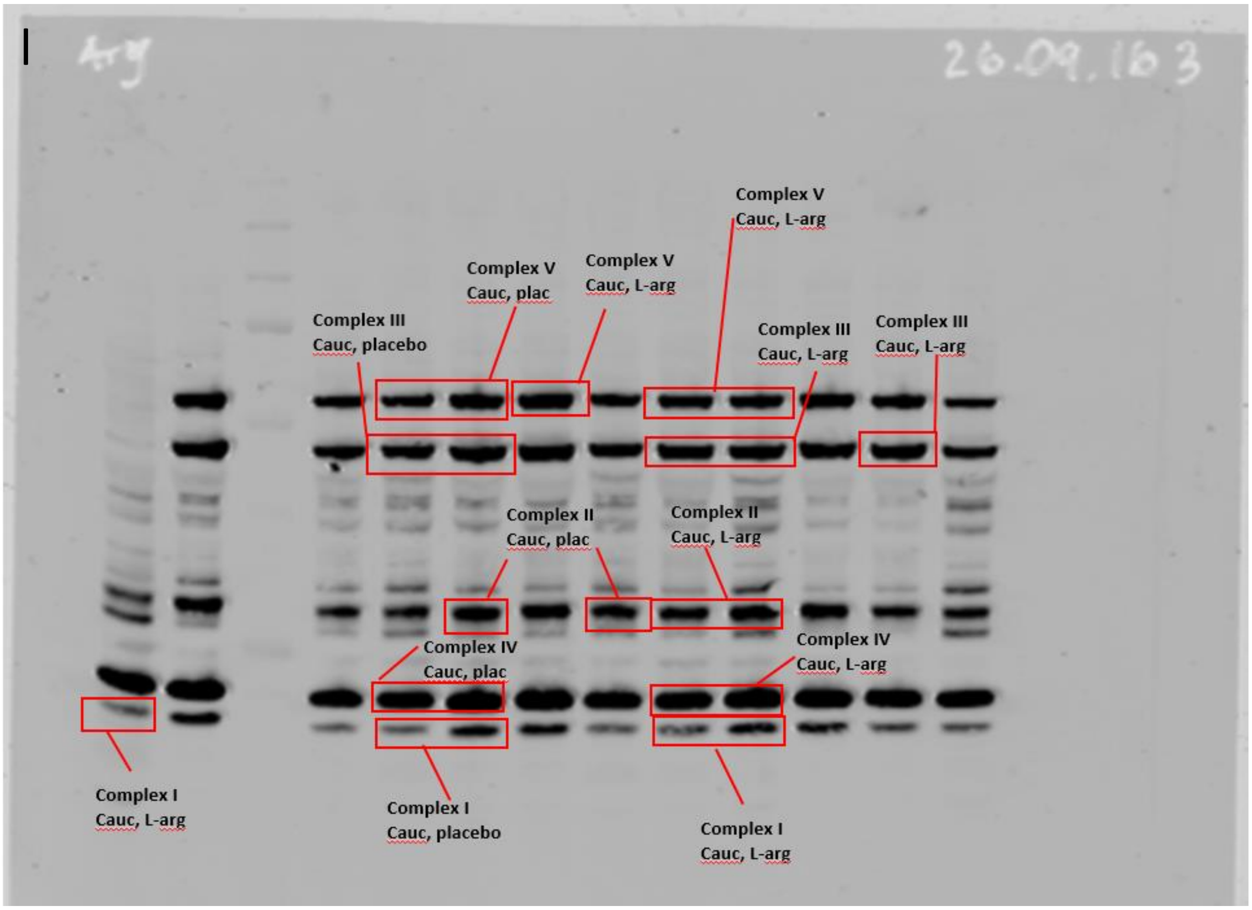
I
Arg
26.09.16 3
Complex V
Cauc, L-arg
Complex V
Cauc, plac
Complex V
Cauc, L-arg
Complex III
Cauc, placebo
Complex III
Cauc, L-arg
Complex III
Cauc, L-arg
Complex II
Cauc, plac
Complex II
Cauc, L-arg
Complex IV
Cauc, plac
Complex IV
Cauc, L-arg
Complex I
Cauc, L-arg
Complex I
Cauc, placebo
Complex I
Cauc, L-arg
